# Numerical Study on the Variability of Plastic CTOD

**DOI:** 10.3390/ma13061276

**Published:** 2020-03-11

**Authors:** Pedro André Prates, Armando Eusébio Marques, Micael Frias Borges, Ricardo Madeira Branco, Fernando Ventura Antunes

**Affiliations:** Centre for Mechanical Engineering, Department of Mechanical Engineering, Materials and Processes (CEMMPRE), University of Coimbra, 3030-788 Coimbra, Portugal; armando.marques@uc.pt (A.E.M.); micaelfriasborges@outlook.pt (M.F.B.); ricardo.branco@dem.uc.pt (R.M.B.); fernando.ventura@dem.uc.pt (F.V.A.)

**Keywords:** CTOD, fatigue, metamodeling, sensitivity analysis, stochastic analysis, variability

## Abstract

This paper presents a numerical study on the influence of material parameters and loading variability in the plastic crack tip opening displacement (CTOD) results. For this purpose, AA7050-T6 was selected as reference material and a middle-cracked tension specimen geometry was considered. The studied input parameters were the Young’s modulus, Poisson’s ratio, isotropic and kinematic hardening parameters and the maximum and minimum applied loads. The variability of the input parameters follows a Gaussian distribution. First, screening design-of-experiments were performed to identify the most influential parameters. Two types of screening designs were considered: one-factor-at-a-time and fractional factorial designs. Three analysis criteria were adopted, based on: main effect, index of influence and analysis of variance. Afterwards, metamodels were constructed to establish relationships between the most influential parameters and the plastic crack tip opening displacement (CTOD) range, based on two types of designs: Face-Centered Central Composite Design and Box-Behnken design. Finally, the metamodels were validated, enabling the expeditious evaluation of the variability in the plastic CTOD range; in addition, the variability in the fatigue crack growth rate was also evaluated.

## 1. Introduction

Fatigue is the main failure mechanism in components submitted to cyclic loads. Design against fatigue based on damage tolerance approach assumes the presence of intrinsic defects, such as cracks. In fact, technological processes like casting, machining, welding and additive manufacturing are known to produce small defects. Accurate assessments of fatigue crack growth (FCG) rates are needed to define the time between inspections. Several models have been proposed in the literature to quantify the FCG rate as a function of loading and/or material parameters (e.g., [1,2,3]). These models are deterministic, i.e., assume that there is a well-defined relation between these parameters and FCG rate. However, the variability of the material, loading, geometry, temperature, etc., introduces uncertainty in the results. In this context, there are two main procedures to overcome the influence of sources of uncertainties [4]: (i) the use of safety factors, which is the conservative solution usually adopted to accommodate uncertainties, and (ii) performing stochastic analysis, which is often supported by probabilistic fatigue models (e.g., [5,6]), metamodeling techniques (e.g., [7,8,9,10,11,12,13]) and more recently by digital twin approaches (e.g., [14,15]). In the context of stochastic fatigue analysis, a probabilistic fracture mechanics approach to predict the fatigue life of aircraft wing attachment bulkheads was proposed by White [16]. A sensitivity analysis was performed to quantify the effect of parameters that significantly influence crack growth in metallic airframes. The studied parameters included: equivalent pre-crack size, crack growth rate, fracture toughness of the material and maximum load per flight. The variability in the total life is most sensitive to the variability in the equivalent pre-crack size, and least sensitive to the variability in the fracture toughness. In other works, FCG tests were performed on cracked specimens made of 2024-T3 aluminum alloy, illustrating the scatter in the FCG rate due to material inhomogeneity [17,18]. A predictive procedure was developed by Bahloul et al. [19], using the finite element method coupled with Monte Carlo, to evaluate the residual FCG life of a 7075-T6 aluminum alloy cracked lug component, considering the stochastic behaviour of the material parameters and the crack tip stress field. The proposed procedure showed a good ability in improving the deterministic FCG life evaluation. An uncertainty quantification methodology was proposed by Sankararaman et al. [7], to predict the crack growth as a function of the number of load cycles in a cylindrical structural component, with complex geometry and subjected to multi-axial loading with variable amplitude. The proposed methodology, which is supported by a Gaussian process metamodel trained via finite element analysis, takes into account sources of uncertainty such as loading conditions and material variability. Wentao et al. [8] proposed an approach to assess the probabilistic life of mixed mode FCG in mechanical components, by coupling finite element analysis and a Kriging-based Monte Carlo metamodel. The use of a Kriging-based metamodel for the probabilistic FCG analysis is shown to be more efficient than the Response Surface Methodology. A probabilistic fatigue life prediction model for multiple site damage in structural components was developed by Kim et al. [9], which resort to an extended finite element method coupled with a Gaussian Process metamodel for computational efficiency. Metamodels were developed by Vélez et al. [10], to relate the crack tip opening displacement (CTOD) with material properties in high-thickness offshore steel welded joints. The studied material properties (hardness, chemical composition, toughness and microstructural morphology) were shown to be significantly related with the CTOD, also being simpler and cheaper to measure and more available than the CTOD.

The aim of this paper is to carry out a numerical analysis on the influence of material parameters and loading variability in the variability of the plastic CTOD results obtained from FCG tests on middle-cracked tension (M(T)) specimens. Antunes et al. [20] developed a material law relating FCG rate with the plastic CTOD (δ_p_). The FCG rate was experimentally measured in standard M(T) samples, while δ_p_ was numerically predicted in models that simulate the experimental work regarding material properties, sample geometry and imposed loadings. It is required a certain amount of crack propagation in the numerical models for each crack length studied, in order to stabilize the cyclic plastic deformation and the crack closure level. The plastic CTOD is measured only at the end of crack propagation, and is not expected to be affected by the crack growth rate imposed artificially in the numerical model. This approach assumes that plastic deformation at the crack tip is the main mechanism behind crack tip and that the plastic CTOD is the crack driving force. Other authors proposed the use of total CTOD [21,22], but Vasco-Olmo et al. [23] proved that the plastic CTOD is the crack driving force. The da/dN–δ_p_ model is supposed to be a material law, which can be used to estimate da/dN in other situations. In this work, the numerical models were changed regarding the material and loading parameters, and the da/dN–δ_p_ model was used to estimate the FCG rate.

Following this introduction, the paper is organized as follows: Section 2 describes the numerical model of the FCG tests; Section 3 presents the results and discussion. Firstly, sensitivity analyses are performed to identify the most influential parameters on the plastic CTOD results. Afterwards, metamodels are constructed with the most influential parameters, to expeditiously evaluate the variability of the plastic CTOD. Finally, the proposed metamodels are validated and compared. Section 4 presents the conclusions.

## 2. Numerical Model

**Figure 1:**
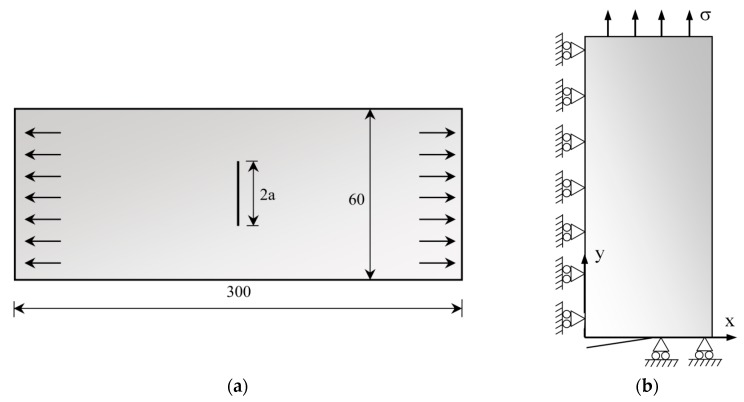
Schematic representation of the M(T) sample: (**a**) Geometry and dimensions (in mm); (**b**) Boundary conditions in the frontal view [20].

Numerical simulations of FCG tests were performed on a standard middle-cracked tension (M(T)) sample geometry, as shown in Figure 1. A straight crack was defined, with an initial size *a*_o_ equal to 5 mm (*a*_o_/W = 0.083). Only 1/8 of the low cycle fatigue test was simulated, due to sample geometry symmetries. Plane strain state was modeled as illustrated in Figure 1b.

The finite element (FE) model of the M(T) sample is composed of 6639 trilinear solid elements, with 13586 nodes. A mesh refinement was performed near the crack tip, using 8 × 8 μm^2^ elements. Crack propagation was simulated by successive debonding of nodes at minimum imposed load. Each crack increment corresponds to one FE and two load cycles were applied before each crack propagation. In each cycle, the crack propagated uniformly over the thickness by releasing both current crack front nodes. 200 load cycles (i.e., 100 crack propagations) were performed, which corresponds to a total crack propagation of Δa = (100 − 1) × 8 μm = 792 μm. The first two load cycles were applied without crack increment, i.e., at a = 5 mm. The aim was the stabilization of crack opening values, since a transient behavior is observed as the residual plastic wake is formed. Additionally, the residual plastic wake may produce crack closure, which affects the crack tip fields. Again, the plastic CTOD is measured at the end of crack propagation and is not expected to be affected by the artificial crack growth rate imposed in the numerical model. The applied remote stresses σ were obtained by dividing the imposed loads by the cross-section area A (= 30 × 0.1 mm^2^).

The numerical simulations were performed with DD3IMP in-house FE solver [24]. The average simulation time for the FCG tests is about 18 h (Intel® Core™i9–7900X 10-Core processor @ 3.3 GHz). The material elastic behavior is considered isotropic and is described by the generalized Hooke’s law, where the Young’s modulus (*E*) and the Poisson’s ratio (*ν*) are the elastic parameters. The plastic behavior is described by the von Mises yield criterion, coupled with the Armstrong-Frederick kinematic hardening law. The von Mises yield criterion is:

(1) $$\sqrt{\frac{3}{2}(\sigma^{\prime }-X^{\prime }):(\sigma^{\prime }-X^{\prime })}-Y_{0}=0$$

where $\sigma^{\prime }$ is the deviatoric Cauchy stress tensor, $X^{\prime }$ is the deviatoric backstress tensor and $Y_{0}$ is the initial yield stress. The Armstrong-Frederick kinematic hardening law is described as follows [25]:

(2) $${\dot{X}}^{\prime }=C_{X}\left[X_{\mathrm{Sat}}\frac{\sigma^{\prime }-X^{\prime }}{\bar{\sigma }}-X^{\prime }\right]{\dot{\bar{\varepsilon }}}^{p}$$

where ${\dot{X}}^{\prime }$ is the backstress rate, representing the translational rate of the center of the yield surface due to plastic deformation, $\bar{\sigma }$ is the equivalent stress and ${\dot{\bar{\varepsilon }}}^{p}$ is the equivalent plastic strain rate; and the material parameters $C_{X}$ and $X_{\mathrm{Sat}}$ represent, respectively, the saturation rate and saturation value of the kinematic hardening (exponential) law.

The material used in this study is a 7050-T6 aluminum alloy, whose elastoplastic cyclic behavior was modeled in a previous work [20]; the values of the material parameters *E*, *ν*, $Y_{0}$, $C_{X}$, $X_{\mathrm{Sat}}$ that describe the elastoplastic behavior of AA7050-T6 were used as reference. In this context, a Gaussian distribution is used for describing the variability of the material parameters and the maximum and minimum imposed loads per cycle, *F*_max_ and *F*_min_. The mean (*μ*) and standard deviation (SD) values of each parameter are detailed in Table 1, where the mean values of the material parameters correspond to the reference ones (see [20]); a coefficient of variation CV = 5% was assumed for each parameter, where CV = SD/*μ*.

**Figure 2:**
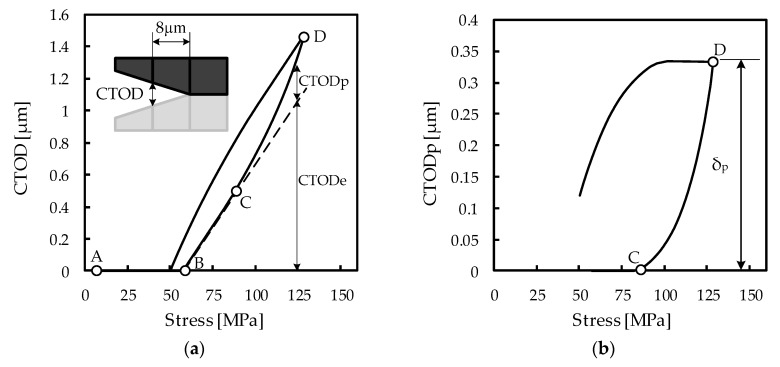
Reference numerical simulation results of AA7050-T6: (**a**) Total crack tip opening displacement (CTOD) vs. applied stress, with schematic representation of CTOD measurement; (**b**) Plastic CTOD vs. applied stress, with indication of δ_p_.

Figure 2a presents the reference numerical simulation results of CTOD vs. applied stress, obtained from the mean values of the input parameters in Table 1 during the 200th load cycle. The CTOD is obtained at the first node at the left of the crack tip (8 μm from the crack tip, as schematized in Figure 2a). At minimum applied stress (point A) the crack is closed, and therefore the CTOD is equal to zero. The increase in the stress value opens the crack at point B. Afterwards, there is a linear region (point B to point C) associated with the material elastic behavior. Plastic deformation initiates after point C, and increases up to the maximum stress at point D. The dashed line shows the elastic CTOD (CTODe); the plastic CTOD (CTODp), is obtained by subtracting the elastic CTOD from the total CTOD. Figure 2b presents the evolution of plastic CTOD with applied stress during the 200th load cycle. Between points C and D, there is a progressive increase of CTODp up to the maximum stress. The range of CTODp, δ_p_, shown in Figure 2b, is assumed to control FCG; accordingly, the variability analysis presented in the subsequent sections will focus on δ_p_.

## 3. Results and Discussion

### 3.1. Sensitivity Analysis

The sensitivity analysis enables excluding the least influential input parameters on the δ_p_ results, to reduce computational effort in the subsequent metamodeling step. For this purpose, numerical simulations were carried out from two types of screening design of experiments (DOE): One-factor-at-a-time (OFAT) and fractional factorial design (FFD) approaches [26]. Three levels were assumed for the input parameters: the mean value (*µ*), the 2.5th (P2.5) and the 97.5th (P97.5) percentiles, which correspond to a 95% confidence interval. Table 2 and Table 3 show the δ_p_ results obtained from the numerical simulations under the OFAT and FFD screening approaches, respectively.

The influence of each input parameter on the plastic CTOD range was quantified using three analysis criteria [26]: (i) Main Effect, (ii) Index of Influence and (iii) Analysis of Variance (ANOVA). This enables assessing the influence of the analysis criterion and screening DOE on the input parameters sensitivity.

The Main Effect of a given input parameter is given by:

(3) $$\mathrm{Main}\;\mathrm{Effect}=\frac{\sum \delta_{p}^{P97.5}-\sum \delta_{p}^{P2.5}}{0.5\times s}$$

where *s* is the total amount of numerical simulations of the screening DOE under analysis, having input parameter values at levels P2.5 and/or P97.5 (i.e., (*s* = 14 for OFAT and *s* = 16 for FFD); $\sum \delta_{p}^{P97.5}$ and $\sum \delta_{p}^{P2.5}$ are the sum of plastic CTOD range values obtained at the 97.5^th^ and the 2.5^th^ percentiles, respectively. According to the Main Effect criterion, the input parameters are assumed influential if the absolute value of their main effect is higher than the average main effect of the seven input parameters, for each DOE.

The Index of Influence of a given parameter is given by:

(4) $$\mathrm{Index}\;\mathrm{of}\;\mathrm{Influence}=\frac{\left|\frac{\sum \delta_{p}^{P2.5}}{0.5\times s}-\delta_{p}^{N}\right|+\left|\frac{\sum \delta_{p}^{P97.5}}{0.5\times s}-\delta_{p}^{N}\right|}{2\times \delta_{p}^{N}}$$

where $\delta_{p}^{N}$ is the nominal value of the plastic CTOD range, and depends on the type of DOE: In case of OFAT, it is obtained from the Simulation 1 in Table 2 (i.e., when considering the mean values of the input parameters shown in Table 1); in case of FFD, it is equal to the average of the δ_p_ values from Table 3. According to the Index of Influence criterion, the input parameters are considered influential when the value of their Index of Influence is higher than the average Index of Influence of the seven input parameters, for each DOE.

ANOVA is used to check the significance of each input parameter, by determining the *p*-value for the F-test at a 95% confidence interval. ANOVA starts with the main effect of each input parameter (Equation (4)), for determining the corresponding sum of squares, SS:

(5) $$\mathrm{SS}=s{\left(\frac{\mathrm{Main}\;\mathrm{Effect}}{2}\right)}^{2}$$

In the next step, the mean of squares, MS, is calculated for each input parameter:

(6) $$\mathrm{MS}=\frac{\mathrm{SS}}{\mathrm{DF}}$$

where DF is the number of degrees of freedom per input parameter, DF = *l* − 1, where *l* is the number of levels of variation considered for each input parameter (excluding the mean, *µ*). Since the number of levels considered is *l* = 2 (i.e., P2.5 and P97.5), then DF = 1. The sum of squares for error, SSE, is:

(7) $$\mathrm{SSE}=\sum_{i=1}^{s}{\left({\delta_{p}}_{i}-{{\hat{\delta }}_{p}}_{i}\right)}^{2}$$

where ${\delta_{p}}_{i}$ is the plastic CTOD range obtained from DOE simulation *i* and ${{\hat{\delta }}_{p}}_{i}$ is the corresponding predicted plastic CTOD range value, obtained by the following linear relationship:

(8) $${{\hat{\delta }}_{p}}_{i}=\alpha_{0}+\alpha_{1}E_{i}+\alpha_{2}\nu_{i}+\alpha_{3}{Y_{0}}_{i}+\alpha_{4}{C_{X}}_{i}+\alpha_{5}{X_{\mathrm{Sat}}}_{i}+\alpha_{6}{F_{\max}}_{i}+\alpha_{7}{F_{\min}}_{i},$$

where *α_j_* are coefficients, with *j* = 0, …, 7. The values of the coefficients were obtained by minimization of Equation (7) resorting to the Generalized Reduced Gradient non-linear optimization algorithm [27]. The mean squared error MSE is given by:

(9) $$\mathrm{MSE}=\frac{\mathrm{SSE}}{s-k-1}$$

where *k* is the number of input parameters under analysis (*k* = 7). The F-ratio is given by:

(10) $$F-\mathrm{Ratio}=\frac{\mathrm{MS}}{\mathrm{MSE}}$$

The F-ratio enables obtaining the *p*-value at a 95% confidence interval. For the 95% confidence interval, the input parameters are assumed influential if the *p*-value is less than 0.05.

The sensitivity analysis results for OFAT and FFD are respectively indicated in Table 4 and Table 5. Whatever the analysis (Main Effect, Index of Influence and ANOVA) and type of screening DOE (OFAT and FFD), the input parameters *E*, *Y*_0_ and *F*_max_ are consistently shown to be the most influential in the δ_p_ results.

### 3.2. Metamodeling

The variability in the δ_p_ results was evaluated resorting to Response Surface Methodology (RSM) metamodels, constructed with the most influential input parameters, *E*, *Y*_0_ and *F*_max_, to reduce the computational effort. In this context, a quadratic polynomial model is adopted to establish the following relationship between *E*, *Y*_0_, *F*_max_ and $\delta_{p}$:

(11) $${\delta_{p}}_{i}^{\mathrm{RSM}}=\beta_{0}+\beta_{1}E_{i}+\beta_{2}{Y_{0}}_{i}+\beta_{3}{F_{\max}}_{i}+\beta_{4}E_{i}{Y_{0}}_{i}+\beta_{5}E_{i}{F_{\max}}_{i}+\beta_{6}{Y_{0}}_{i}{F_{\max}}_{i}+\beta_{7}E_{i}^{2}+\beta_{8}{Y_{0}}_{i}^{2}+\beta_{9}{F_{\max}}_{i}^{2}$$

where ${\delta_{p}}_{i}^{\mathrm{RSM}}$ is the value of plastic CTOD range predicted by the RSM model for RSM simulation *i* and *β_j_* are fitting RSM coefficients, with *j* = 0, …, 9. Equation (11) can be also written as a system of linear equations, as follows:

(12) $${\delta_{p}}_{i}=H_{ij}\beta_{j}+\varepsilon_{i}$$

where ${\delta_{p}}_{i}$ (*i* = 1, …, *n*) is the vector of plastic CTOD range measurements obtained from *n* RSM simulations, $\varepsilon_{i}$ is an error term and $H_{ij}$ is the linear system matrix,

(13) $$H_{ij}=\left[\begin{array}{cccccccccc} 1 & E_{1} & {Y_{0}}_{1} & {F_{\max}}_{1} & E{Y_{0}}_{1} & E{F_{\max}}_{1} & Y_{0}{F_{\max}}_{1} & E_{1}^{2} & {Y_{0}}_{1}^{2} & {F_{\max}}_{1}^{2}\\ \vdots & \vdots & \vdots & \vdots & \vdots & \vdots & \vdots & \vdots & \vdots & \vdots \\ 1 & E_{n} & {Y_{0}}_{n} & {F_{\max}}_{n} & E{Y_{0}}_{n} & E{F_{\max}}_{n} & Y_{0}{F_{\max}}_{n} & E_{n}^{2} & {Y_{0}}_{n}^{2} & {F_{\max}}_{n}^{2} \end{array}\right]$$

**Figure 3:**
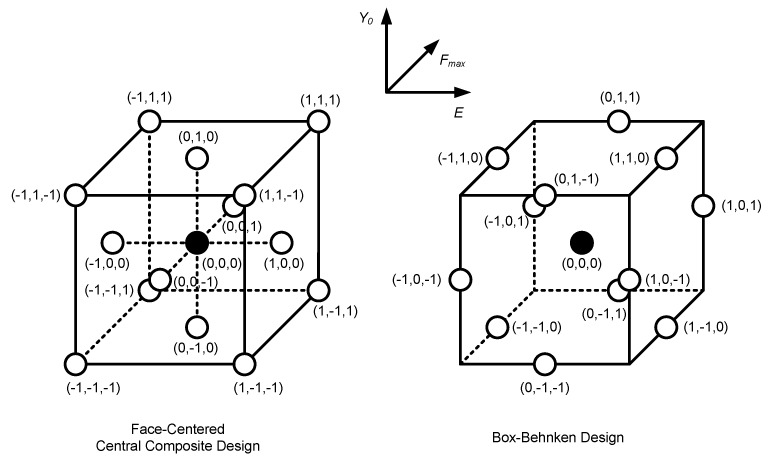
Face-Centered Central Composite Design (FCCCD) and Box-Behnken design (BBD) design points for constructing Response Surface Methodology (RSM) metamodels.

RSM simulations were performed based on two types of designs: Face-Centered Central Composite Design (FCCCD) and Box-Behnken design (BBD); the aim is to assess the influence of the choice of RSM metamodel on predicting the variability in the δ_p_ results. Figure 3 shows the map of points (i.e., sets of input parameters) considered in the FCCCD and BBD design space, each point corresponding to one numerical simulation. Three levels of variation are considered for each input parameter: P2.5, *µ* and P97.5 (see Table 1); these levels are referred in Figure 3 as −1, 0, 1, respectively. The FCCCD design uses vertex points to assess the plastic CTOD range under extreme combinations between the three input parameters; as such, the resulting response surface is expected to describe extreme (and highly unlikely) behavior more accurately than that obtained using Box-Behnken (which lacks vertex points, having edge points instead). On the other hand, the Box-Behnken design is a computationally cheaper alternative to FCCCD, requiring fewer numerical simulations. The RSM numerical simulations assumed the mean values (*µ*) concerning the least influential parameters (*ν*, *C*_X_, *X*_Sat_ and *F*_min_—see Table 1).

Table 6 presents the least-squares solution for the RSM coefficients associated with the FCCCD and BBD designs. Whatever the design, FCCCD or BBD, the number of numerical simulations *n* is greater than the number of RSM coefficients *β* (i.e., *n* > *j*); in this case, the least-squares solution is the one that minimizes the sum of squared error at the design points, $\varepsilon_{i}=\sqrt{{{{(\delta }_{p}}_{i}-H_{ij}\beta_{j})}^{2}}$, given by [28]:

(14) $$\beta_{j}={\left(H_{ji}H_{ij}\right)}^{-1}H_{ji}{\delta_{p}}_{i}$$

Table 7 and Table 8 compare the δ_p_ responses obtained from the FCCCD or BBD simulations with those predicted by the corresponding RSM metamodels; the metrics R^2^ and relative root mean squared error (RRMSE) were chosen to quantify the fitting performance of the design points. In general, the obtained metamodels are able to adequately describe the AA7050-T6 plastic CTOD range measurements at the design points; also, the metamodel accuracy is best when considering the Box-Behnken design (R^2^ = 0.9996, RRMSE = 0.4%) than FCCCD (R^2^ = 0.9927, RRMSE = 2.0%).

### 3.3. Metamodel Validation

**Figure 4:**
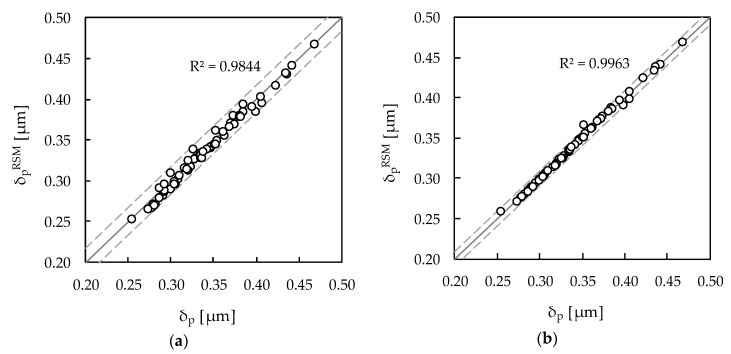
Correlation between the δ_p_ values obtained by random numerical simulations and those predicted by the response surfaces constructed with (**a**) FCCCD and (**b**) BBD simulations.

The R^2^ analysis shown in Table 7 and Table 8 enabled a first assessment of the predictive ability of the RSM metamodels. At this stage, 60 random numerical simulations are performed to check if the response surfaces still represent a proper approximation of the plastic CTOD range within the range of variation of the main input parameters. Figure 4 presents the correspondence between numerical simulation and response surface δ_p_ results, for the FCCCD and BBD based metamodels. In this figure, each point corresponds to one numerical simulation. The scatter of the random points around the straight line is assessed with R^2^; the straight line indicates the ideal metamodel, in which the predicted response is equal to that obtained by numerical simulation. In general, both FCCCD and BBD metamodeling approaches provide accurate predictions of δ_p_, with RRMSE equal to 1.7% (FCCCD) and 0.9% (BBD), although dispersion is more evident for FCCCD (with R^2^ = 0.9844—see Figure 4a) than for BBD (with R^2^ = 0.9963—see Figure 4b). This corroborates the adequacy of the proposed RSM metamodels for establishing the relationship between the most influential inputs and δ_p_.

### 3.4. Variability Analysis Based on Metamodels

**Figure 5:**
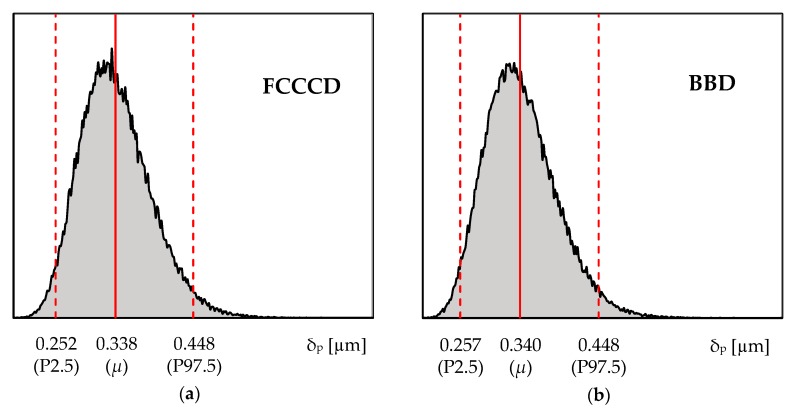
Plastic CTOD range histograms generated from 100,000 random sets of input parameters using (**a**) FCCCD and (**b**) BBD metamodels. The mean δ_p_ values and the 2.5th and 97.5th percentiles are also represented.

The RSM metamodels are now used to expeditiously predict the variability in the δ_p_ results. Accordingly, 100,000 random sets of input parameters *E*, *Y*_0_ and *F*_max_ were generated. The values of each set of input parameters were used as input for the RSM models (i.e., see Equation (11), with the values of the RSM coefficients shown in Table 6), to predict the δ_p_ variability. Figure 5 presents the δ_p_ variability results predicted by the RSM metamodels based on FCCCD and BBD designs. Both histograms present the predicted mean δ_p_ values as well as percentiles P2.5 and P97.5, enabling the definition of a 95% confidence interval. According to the figure, the RSM metamodels predict the variability in the plastic CTOD range in a similar way, both presenting right-skewed distributions. Such skewed distributions contrast with the normal distribution, which was assumed for describing the variability of the input parameters.

Table 9 presents descriptive statistics of the δ_p_ histograms shown in Figure 5. The predicted mean δ_p_ value (approx. 0.340 µm) is close to the δ_p_ value obtained from the reference simulation (=0.334 µm, see Simulation 1 in Table 1), which is assumed to be the exact solution under a deterministic analysis. However, from the perspective of the current stochastic analysis, it is possible to estimate not only the mean δ_p_ value, but also the associated variance; accordingly, the predicted variability in the δ_p_ results follows a coefficient of variation close to 15% (note that the variability in the input parameters follows *σ*/*µ* = 5%). This emphasizes the advantage and the importance of stochastic analysis, in detriment of deterministic analysis, which is key in the robust design and optimization for fatigue life of critical components subjected to time-varying loading.

Additionally, the following linear relationship between plastic CTOD range (δ_p_, in µm) and FCG rate (da/dN, in µm/cycle) from a previous work by Antunes et al. [20] allows predicting the variability in terms of da/dN:

(15) $$\mathrm{da}/\mathrm{dN}=0.5246\times \delta_{p}$$

According to Equation (15), the predicted mean value for da/dN is close to 0.178 µm/cycle, with a coefficient of variation close to 15%. It should be highlighted that the variability analysis results may depend on the assumptions considered in the numerical model, such as the specimen geometry, number of loading cycles between crack propagations, boundary conditions, type of material, among others. This motivates further numerical studies on plastic CTOD range variability. However, linear elastic fracture parameters are commonly adopted to predict da/dN, although fatigue crack propagation is closely linked with plastic deformation occurring at the crack tip. This motivates conducting variability studies focused on the stress intensity factor range ΔK, for comparison purposes with δ_p_.

## 4. Conclusions

This paper concerns a numerical study on the influence of material parameters and loading variability in the plastic CTOD range of AA7050-T6 on M(T) specimens. First, the most influential input parameters were identified based on the analysis of two types of screening DOE approaches (OFAT and FFD), according to three analysis criteria (Main Effect, Index of Influence and ANOVA). Afterwards, RSM metamodels were established to predict the variability in the δ_p_ results in an expeditious way, based on two types of designs (FCCCD and BBD). Additionally, the variability in the FCG rate, da/dN, was also predicted, based on da/dN–δ_p_ relationships previously established in a previous work by the authors. The following remarks can be drawn:

- The type of screening DOE and analysis does not interfere with the identification of the relevant parameters influencing the plastic CTOD range: the parameters *E*, *Y*_0_ and *F*_max_ are consistently shown to be the most influential;
- Both FCCCD and BBD metamodeling approaches provide similar and accurate predictions of the plastic CTOD range, with RRMSE = 1.7% (FCCCD) and RRMSE = 0.9% (BBD);
- The predicted variability in the plastic CTOD range results presents right-skewed distributions that follow a coefficient of variation close to 15%.

## Figures and Tables

**Table 1:** Mean (*μ*) and standard deviation (SD) values of the studied input parameters; percentiles 2.5th (P2.5) and 97th (P97.5) are also shown.

AA7050-T6	*E* (GPa)	*ν*	*Y*_0_ (MPa)	*C*_X_	*X*_Sat_ (MPa)	*F*_max_ (N)	*F*_min_ (N)
*µ*	71.70	0.3300	420.50	228.91	198.35	385.29	19.26
SD	3.59	0.0165	21.03	11.45	9.92	19.26	0.96
P2.5	64.67	0.2977	379.29	206.48	178.91	347.53	17.37
P97.5	78.73	0.3623	461.71	251.34	217.79	423.05	21.15

**Table 2:** Numerical simulation results of δ_p_ for the one-factor-at-a-time (OFAT) screening design.

Simulation	*E* (GPa)	*ν*	*Y*_0_ (MPa)	*C*_X_	*X*_Sat_ (MPa)	*F*_max_ (N)	*F*_min_ (N)	δ_p_ (µm)
1	*µ*	*µ*	*µ*	*µ*	*µ*	*µ*	*µ*	0.334
2	P2.5	*µ*	*µ*	*µ*	*µ*	*µ*	*µ*	0.364
3	P97.5	*µ*	*µ*	*µ*	*µ*	*µ*	*µ*	0.304
4	*µ*	P2.5	*µ*	*µ*	*µ*	*µ*	*µ*	0.340
5	*µ*	P97.5	*µ*	*µ*	*µ*	*µ*	*µ*	0.326
6	*µ*	*µ*	P2.5	*µ*	*µ*	*µ*	*µ*	0.379
7	*µ*	*µ*	P97.5	*µ*	*µ*	*µ*	*µ*	0.303
8	*µ*	*µ*	*µ*	P2.5	*µ*	*µ*	*µ*	0.337
9	*µ*	*µ*	*µ*	P97.5	*µ*	*µ*	*µ*	0.326
10	*µ*	*µ*	*µ*	*µ*	P2.5	*µ*	*µ*	0.341
11	*µ*	*µ*	*µ*	*µ*	P97.5	*µ*	*µ*	0.322
12	*µ*	*µ*	*µ*	*µ*	*µ*	P2.5	*µ*	0.264
13	*µ*	*µ*	*µ*	*µ*	*µ*	P97.5	*µ*	0.421
14	*µ*	*µ*	*µ*	*µ*	*µ*	*µ*	P2.5	0.335
15	*µ*	*µ*	*µ*	*µ*	*µ*	*µ*	P97.5	0.333

**Table 3:** Numerical simulation results of δ_p_ for the fractional factorial design (FFD) screening design.

Simulation	*E* (GPa)	*ν*	*Y*_0_ (MPa)	*C*_X_	*X*_Sat_ (MPa)	*F*_max_ (N)	*F*_min_ (N)	δ_p_ (µm)
1	P2.5	P2.5	P2.5	P2.5	P2.5	P2.5	P2.5	0.346
2	P97.5	P2.5	P2.5	P2.5	P97.5	P2.5	P97.5	0.273
3	P2.5	P97.5	P2.5	P2.5	P97.5	P97.5	P2.5	0.529
4	P97.5	P97.5	P2.5	P2.5	P2.5	P97.5	P97.5	0.499
5	P2.5	P2.5	P97.5	P2.5	P97.5	P97.5	P97.5	0.418
6	P97.5	P2.5	P97.5	P2.5	P2.5	P97.5	P2.5	0.366
7	P2.5	P97.5	P97.5	P2.5	P2.5	P2.5	P97.5	0.269
8	P97.5	P97.5	P97.5	P2.5	P97.5	P2.5	P2.5	0.220
9	P2.5	P2.5	P2.5	P97.5	P2.5	P97.5	P97.5	0.561
10	P97.5	P2.5	P2.5	P97.5	P97.5	P97.5	P2.5	0.425
11	P2.5	P97.5	P2.5	P97.5	P97.5	P2.5	P97.5	0.492
12	P97.5	P97.5	P2.5	P97.5	P2.5	P2.5	P2.5	0.270
13	P2.5	P2.5	P97.5	P97.5	P97.5	P2.5	P2.5	0.273
14	P97.5	P2.5	P97.5	P97.5	P2.5	P2.5	P97.5	0.232
15	P2.5	P97.5	P97.5	P97.5	P2.5	P97.5	P2.5	0.421
16	P97.5	P97.5	P97.5	P97.5	P97.5	P97.5	P97.5	0.324

**Table 4:** Main Effect, Index of Influence and ANOVA results obtained from the OFAT simulations (see Table 2). The values in bold indicate that the respective parameters are assumed influential.

OFAT	*E* (GPa)	*ν*	*Y*_0_ (MPa)	*C*_X_	*X*_Sat_ (MPa)	*F*_max_ (N)	*F*_min_ (N)
Main Effect	0.0598	0.0133	0.0752	0.0109	0.0192	0.1561	0.0016
Index of Influence	0.0895	0.0199	0.1125	0.0164	0.0287	0.2335	0.0024
ANOVA *p*-value	0.0010	0.2309	0.0003	0.3144	0.1020	0.0000	0.8778

**Table 5:** Main Effect, Index of Influence and ANOVA results obtained from the FFD simulations (see Table 3). The values in bold indicate that the respective parameters are assumed influential.

FFD	*E* (GPa)	*ν*	*Y*_0_ (MPa)	*C*_X_	*X*_Sat_ (MPa)	*F*_max_ (N)	*F*_min_ (N)
Main Effect	0.0874	0.0162	0.1087	0.0101	0.0014	0.1460	0.0270
Index of Influence	0.1182	0.0219	0.1469	0.0136	0.0020	0.1974	0.0365
ANOVA *p*-value	0.0087	0.5039	0.0031	0.6751	0.9516	0.0007	0.2815

**Table 6:** Least-squares solutions of RSM coefficients obtained from the FCCCD and BBD designs.

RSM Coefficients	Face-Centered Central Composite Design	Box-Behnken Design
*β*_0_	3.061 × 10^0^	1.814 × 10^−1^
*β*_1_	−3.124 × 10^−2^	−6.938 × 10^−2^
*β*_2_	−6.633 × 10^−3^	−7.472 × 10^−4^
*β*_3_	−1.783 × 10^−3^	2.630 × 10^−3^
*β*_4_	3.220 × 10^−5^	9.827 × 10^−6^
*β*_5_	−7.202 × 10^−6^	−3.067 × 10^−5^
*β*_6_	−6.909 × 10^−6^	−1.082 × 10^−5^
*β*_7_	1.158 × 10^−4^	7.009 × 10^−5^
*β*_8_	7.281 × 10^−6^	3.872 × 10^−6^
*β*_9_	9.765 × 10^−6^	8.157 × 10^−6^

**Table 7:** Comparison between δ_p_ results obtained from the FCCCD simulations with those predicted by the RSM metamodel; the respective R^2^ value is also shown.

Simulation	*E* (GPa)	*Y*_0_ (MPa)	*F*_max_ (N)	δ_p_ (µm)	δ_p_^RSM^ (µm)	
1	P2.5	P2.5	P2.5	0.323	0.334	R^2^ = 0.9927 RRMSE = 2.0%
2	P97.5	P2.5	P2.5	0.270	0.264	
3	P2.5	P97.5	P2.5	0.271	0.265	
4	P97.5	P97.5	P2.5	0.225	0.233	
5	P2.5	P2.5	P97.5	0.543	0.534	
6	P97.5	P2.5	P97.5	0.452	0.457	
7	P2.5	P97.5	P97.5	0.418	0.423	
8	P97.5	P97.5	P97.5	0.395	0.383	
9	P2.5	*µ*	*µ*	0.364	0.363	
10	P97.5	*µ*	*µ*	0.304	0.308	
11	*µ*	P2.5	*µ*	0.379	0.378	
12	*µ*	P97.5	*µ*	0.303	0.307	
13	*µ*	*µ*	P2.5	0.264	0.256	
14	*µ*	*µ*	P97.5	0.421	0.431	
15	*µ*	*µ*	*µ*	0.334	0.330	

**Table 8:** Comparison between δ_p_ results obtained from the Box-Behnken simulations with those predicted by the RSM metamodel; the respective R^2^ value is also shown.

Simulation	*E* (GPa)	*Y*_0_ (MPa)	*F*_max_ (N)	δ_p_ (µm)	δ_p_^RSM^ (µm)	
1	P2.5	P2.5	*µ*	0.417	0.419	R^2^ = 0.9996 RRMSE = 0.4%
2	P97.5	P2.5	*µ*	0.348	0.349	
3	P2.5	P97.5	*µ*	0.335	0.334	
4	P97.5	P97.5	*µ*	0.277	0.276	
5	P2.5	*µ*	P2.5	0.293	0.292	
6	P97.5	*µ*	P2.5	0.244	0.244	
7	P2.5	*µ*	P97.5	0.471	0.471	
8	P97.5	*µ*	P97.5	0.389	0.391	
9	*µ*	P2.5	P2.5	0.294	0.293	
10	*µ*	P97.5	P2.5	0.246	0.248	
11	*µ*	P2.5	P97.5	0.493	0.490	
12	*µ*	P97.5	P97.5	0.378	0.378	
13	*µ*	*µ*	*µ*	0.334	0.334	

**Table 9:** Descriptive statistics of the δ_p_ histograms shown in Figure 5.

δ_p_	FCCCD-Based Model	BBD-Based Model
Mean value, µ	0.338 µm	0.340 µm
Standard deviation, SD	0.050 µm	0.049 µm
Coefficient of variation, CV	14.9%	14.5%
2.5th percentile, P2.5	0.252 µm	0.257 µm
97.5th percentile, P97.5	0.448 µm	0.448 µm
